# An open source ultrasonic flowmeter for monitoring the input/output flow rates of wastewater treatment plants

**DOI:** 10.1016/j.ohx.2024.e00613

**Published:** 2024-12-11

**Authors:** Hélène Guyard, Stéphanie Prost-Boucle, Julien Sudre, Sylvain Moreau, Arnold Imig, Gabrielle Favreau, Valerie Quatela, Remi Clement

**Affiliations:** a Univ. Grenoble Alpes, IRD, CNRS, INRAE, Grenoble INP*, IGE, 38000 Grenoble, France *Institute of Engineering and Management, Univ. Grenoble Alpes, France; b INRAE - French National Research Institute for Agriculture, Food and Environment, REVERSAAL Research Unit, 5 rue de la Doua, CS 20244, 69625 Villeurbanne Cedex, France; c INRAE - French National Research Institute for Agriculture, Food and Environment, PROSE Research Unit, 1 rue Pierre Gilles de Gennes, 92160 Antony, France

**Keywords:** Wastewater, Treatment plant, Monitoring, Water level, Ultrasonic sensor, Low-tech

## Abstract

Sensors play an important role in both the continuous monitoring and intermittent analyses, which are essential for the study of wastewater treatment plant management and conducting related research. Given the significant environmental impact of the issues involved, accurate measurement of the volume of water flowing into and out of treatment plants is a key parameter for plant management, ecotoxicological studies and academic research programs. Traditionally, flow measurements have been based on calibrated weirs or venturi flumes, using water level measurements for conversion into flow, according to established relationships. In this article, the authors propose an innovative approach to explore the feasibility of developing an open-source, reparable and cost-effective data logger for flow monitoring using ultrasonic technology. By leveraging Arduino modules and a complementary Grove shield, the proposed data logger offers seamless integration and affordability. In particular, it features an on-board web server to facilitate data collection and device testing, offering accessibility through Wi-Fi connectivity with smartphones or computers. The authors demonstrate the effectiveness of their flowmeter by comparing its performance with that of a commercial reference flowmeter, yielding a maximum permissible measurement error of 0.6 mm on the water level measurement. Furthermore, they demonstrate the durability and reliability of the developed data logger through extensive field-testing over a 9-month period.

## Graphical abstract

**Figure:**
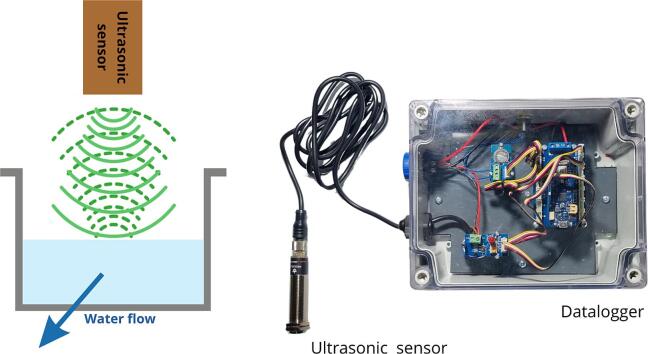

**Table of specifications:** 

Hardware name	Setier-data logger-flowmeter
Subject area	Environmental, planetary and agricultural sciences
Hardware type	Field measurements and sensors
Closest commercial analog	Ultrasonic flowmeter
Open-source license	CERN-OHL-P
Cost of hardware	< €420 for all electronic parts
Source file repository	https://doi.org/10.17605/osf.io/t9fzs

## 1 Hardware in context

Various types of sensors play a crucial role in both continuous and intermittent operations within urban water management systems. These sensors are essential tools for monitoring and controlling medium-sized (> 10,000 people equivalent − PE) and large-scale (> 100,000 PE) treatment facilities [1]. They enable the real-time recording of essential physical parameters such as inflow and outflow rates, as well as temperature variations within the infrastructure [2]. Additionally, they provide continuous measurements of parameters specifically tailored for monitoring physicochemical processes throughout the treatment stages, including electrical conductivity, pH, turbidity, redox potential, dissolved oxygen in basins [3] and airflow production for oxidation treatment [4].

In smaller treatment plants (< 2,000 PE), the scope of water quality and quantity monitoring is often more limited. The monitoring is frequently restricted to batch counting or the utilisation of malfunction sensors designed to detect issues such as effluent overflow [5]. Moreover, when considering extensive systems like vertical flow constructed wetlands (VFCW), valued for their operational simplicity, integrating sensor technologies may be perceived as potentially complicating a system primarily designed for rural settings [6].

The feasibility of implementing sensor technologies depends heavily on factors such as the specific parameters to be measured, acquisition costs and ongoing maintenance costs. Local authorities and operators often find the acquisition and maintenance of sensor networks financially burdensome, particularly in smaller facilities where budgets are constrained [7]. The complexity of repairing and maintaining these sensor solutions, which are often perceived as opaque to end users, adds to the challenges [8]. Upgrading these systems tends to require a complete overhaul at regular intervals, thus further adding to operational costs and logistical complexity [9].

The use of open source and open hardware solutions for monitoring water treatment facilities remains largely unexplored due to a lack of context-specific solutions. A crucial parameter in sewage treatment plant monitoring is the continuous tracking of inlet and outlet flow rates [10]. Measuring volumes passing through a wastewater treatment plant is a mandatory parameter, whatever the size of the facility. As such, flow measurement is governed by regulatory requirements and is subject to normalisation. Traditional methods of monitoring these assets typically rely on commercial systems using radar or ultrasonic technologies. Such systems work by calculating flow rates using calibrated channels or pipes, with sensors converting water level measurements into flow rates using manufacturer-provided algorithms [11].

The use of open source and open hardware systems is common in various environmental fields: seismology [12], air quality [13], geophysics [14] and hydrology [15]. However these technologies are still relatively novel for specific applications such as biogas production [16] or water analysis and treatment. Research and development in these areas are just now beginning to emerge [17]. For wastewater treatment, one main limitation encountered in this context is the presence of highly contaminated water, which can lead to clogging or damage of the sensors used to measure various water parameters. Indeed, such a harsh environment presents significant challenges for the durability and accuracy of measurement instruments. Despite these challenges, significant advances are underway.

The first initiatives have sought to deploy data acquisition systems based on the open source and open hardware technologies. These systems are designed to be both accessible and customisable, hence enabling their adaptation to the specific needs of water treatment. They are modular, as they are designed and adapted specifically for the intended use: their design can be adapted to the location in which the low-cost measuring device is to be installed, just as the sensor(s) will be chosen for their performance and measurement uncertainties in line with the type of water to be analysed [17]. Additionally, integrating low-cost sensors into these systems represents a promising solution to make water monitoring technologies more economical and accessible [8], [18], [19], [20].

Recent studies have shown promising progress in open source flow measurement solutions. The article by Daria Wotzka et *al.* [21] presents an Arduino-based system with ultrasonic sensors for flow measurement in open channels, achieving a complete solution for less than 100€. Similarly, Lucas Gobatti et *al.* [22] have developed a low-cost flow measurement system specifically designed for open channels in wastewater treatment plants, with a particular focus on small-scale facilities. These recent publications highlight the growing interest and viability of open hardware and open-source device for flow monitoring applications. Our work differs from these previous studies by proposing a seamless tool specifically adapted for small-sized open channels, making it even more accessible for small treatment plant operators and more reparable.

The main objective of our article is to present an open source ultrasonic flowmeter with all the procedures described to seamlessly design the construction of this equipment by operating personnel who do not have the necessary skills to install and maintain these sensors [17]. This innovative device presented in this article is designed to track variations within reference channels encompassing a water-level range of 0 to 1.5 m, as a means of catering to both research and operational applications. A key parameter is the monitoring taking place in both the inlet and outlet flow rates of the system, whether for research or for facility operators. To the best of our knowledge, there is no detailed documentation on the step-by-step design of an open source flowmeter suitable for measuring flows in channels in wastewater treatment plants. The experiment is fully documented here, to enable an operator to purchase the necessary parts, and assemble the sensor along with the associated data logger as simply as possible. Comprehensive, user-friendly documentation of this type of low-tech equipment is essential [17], [18] to reduce the obstacles often cited to the deployment of the technology (limiting the need for electronics skills, reducing the device's energy consumption, offering a simple DIY option…) for use both in scientific research and in water treatment facilities. Our article highlights an open source ultrasonic flowmeter, installed without soldering and capable of being repaired. This flowmeter will allow tracking variations in reference channels, including water-level measurements from 0.1 to 1.5 m.

## 2 Hardware description

### 2.1 Principle of measurement

**Fig. 1:**
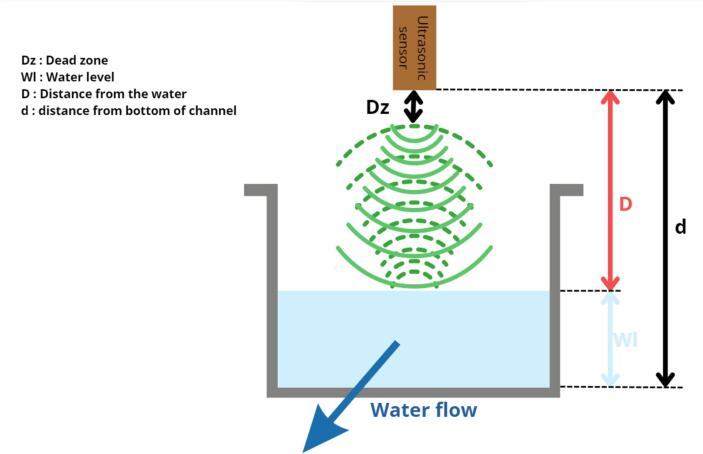
Operating principle of the ultrasonic sensor (ultrasonic waves shown in green).

The principle of measurement is presented in Fig. 1. An ultrasonic sensor is a device that uses ultrasonic waves to measure distance. Sound waves travel at known speeds through the air, depending both on air temperature and humidity. When encountering an obstacle (either solid or liquid), the ultrasonic waves bounce off the obstacle and return to the emission source. By measuring the time between the emission of an ultrasonic wave and its return to the sensor, it is possible to determine the distance D between the sensor and the obstacle, water in our case (Eq. (1):

(1) D=V.t/2

where V(m/s) is the velocity of the ultrasonic wave in the air, t (s) the time between the emission and reception, and D (m) is the distance from the water.

In our case study, the ultrasonic sensor is placed above a channel of known cross-section, at a minimum distance from the water level that is at least greater than the sensor's measurement “dead zone”. The distance D measured by the sensor is then subtracted from the fixed distance d corresponding to the position of the ultrasonic emission surface and the bottom of the channel in order to calculate the water level W_L_ Eq. (2).

(2) Wl=d-D

A temperature correction is applied directly to the measurement, thanks to a thermometer integrated into the ultrasonic transducer. Once this correction has been applied, the flow rate can be calculated from the measured water level, using an equation that depends on the open channel used.

### 2.2 Description

The proposed system is a device designed to measure the flow in a reference channel, which can be a calibrated Venturi type channel or behind spillway thresholds. These measurements are adapted to water level variations observed in wastewater treatment plants for such structures, with variations ranging from 0 to 1.5 m. The water level is measured using the ultrasonic probe DFROBOT, URM14, SEN358, 200 kHz. The simple and robust design makes it easy to replace faulty components without having to replace the entire data logger, thereby ensuring a long service life.

**Fig. 2:**
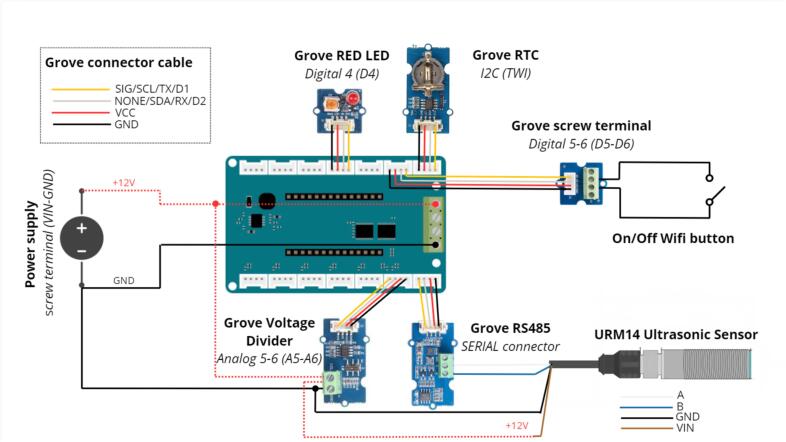
Schematic design of the flowmeter.

**Fig. 3:**
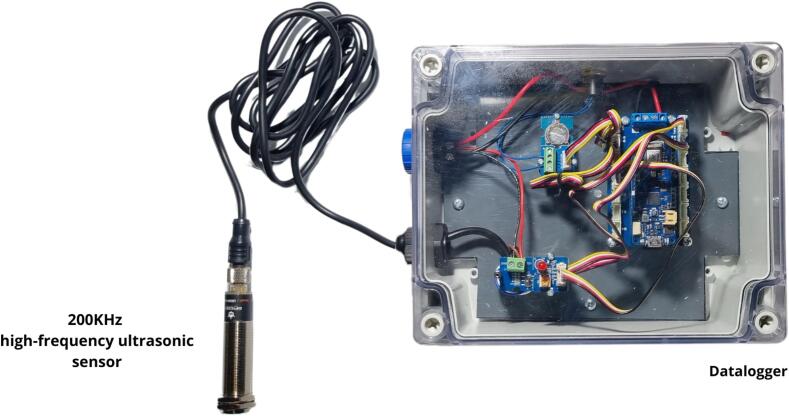
The flowmeter prototype.

An Arduino MKR WIFI 1010 board was used for the data logger design. This board has the advantage of directly integrating a Wi-Fi module. An MKR SD board is added to the previous one for data storage. To avoid soldering, we used an Arduino MKR Carrier board. Grove connectors were employed to facilitate the connection of the components needed for the data logger and sensors (Fig. 2). The main feature of our system is its integration of a web server. This makes it possible to visualise, via Wi-Fi, the main data of the data logger and the configuration of the flowmeter according to the selected channel shape. Data files can also be retrieved by Wi-Fi connection. The implementation of a dedicated web server simplifies use and data collection: no installation is required on a phone or computer, just connect to the Wi-Fi network and retrieve data via the web interface. Compared to commercial tools, our device stands out for its repairability and freedom to set parameters offered to users. In addition, the data logger can be used in various research applications, such as urban hydrology or river water level measurements, by means of other ultrasonic sensors. Direct connection to a Wi-Fi or LoRaWAN network is also feasible (not yet implemented). While this article focuses on presenting an ultrasonic flowmeter (Fig. 3), the data logger employed can easily be adapted for measuring other parameters.

### 2.3 Highlights of this project

• Moderate cost, with a high level of repairability;
• Assembly without the use of a soldering iron;
• Ease to use;
• No software to install, data can be downloaded from the data logger’s web server;
• Adaptable to other water-level measurement applications.

## 3 Summary of the design files

Table 1 lists the relevant design files for the flowmeter device. The type of file is indicated in the second column of the table. The file categories are a 3D printed hardware (STL) file, and a Figure (pdf) file.

**Table 1:** Design file summary.

Design file name	File type	Open source license	Location of the file
CAD_3DPrint_support_flowmeter.STL	STL File	CC BY 4.0	https://doi.org/10.17605/osf.io/t9fzsFolder: support_design_file
Flowmeter_data_logger_hole.pdf	PDF files, Footprint for cutting Support	CC BY 4.0	https://doi.org/10.17605/osf.io/t9fzsFolder: support_design_file

## 4 Bill of material summary

The complete ultrasonic flowmeter system can be assembled using 28 distinct components with a total cost of approximately 406€. The complete set of components is listed in Table 2.

**Table 2:** List of materials with reference and price.

	Designator	Component	Number	Cost per unit −currency	Total cost −currency	Source of materials	Material type
1	Microcontroller	Arduino MKR Wi-fi 1010	1	35.90€	35.90€	*Link_1*	Electronic
2	SD card module	Arduino MEM Shield	1	21.76€	21.76€	*Link_2*	Electronic
3	Carrier for Arduino MKR	Arduino MKR Connector Carrier	1	21.76€	21.76€	*Link_3*	Electronic
4	Clock	RTC DS 1307	1	6.90€	6.90€	*Link_4*	Electronic
5	Shield grove RS485	RS485 Grove	1	5.32€	5.32€	*Link_20*	Electronic
6	Voltage divider	Grove Voltage divider	1	7.48€	7.48€	*Link_5*	Electronic
7	Red LED	Red LED	1	1.67€	1.67€	*Link_21*	Electronic
8	Analogic connector	Grove Screw Terminal	1	2.05€	2.05€	*Link_7*	Electronic
9	Enclosure	Box Polycarbonate 201x163x98mm IP65	1	38.41€	38.41€	*Link_18*	Plastic
10	Spacer	Spacer M2 x 20 mm	12	0.834€	10.01€	*Link_12*	Metal
11	Spacer	Spacer M3 x 20 mm	4	1.17€	4.68€	*Link_13*	Metal
12	Nut	Nut M3	250^1^	7.78€	7.78€	*Link_16*	Metal
13	Nut	Nut M2	250^2^	7.05€	7.05€	*Link_17*	Metal
14	Screw	Screw M3x8mm	100^3^	4.08€	4.08€	*Link_15*	Metal
15	Screw	Screw M2x8mm	100^4^	4.26€	4.26€	*Link_14*	Metal
16	Pushbutton	Pushbutton	1	7.71€	7.71€	*Link_22*	Electronic
17	Sensor	Ultrasonic Sensor, URM14, SEN0358	1	92.07€	92.07€	*Link_19*	Electronic
18	Cable gland	Cable gland 12 mm hole	1^5^	5.70€	5.70€	Link_28	Plastic
19	Female panel connector	Female panel connector	1	23.88€	23.88€	*Link_9*	Electronic
20	Male cable connector	Male cable connector	1	25.12€	25.12€	*Link_10*	Electronic
21	Wire	Black wire 1-mm^2^ section	1^6^	50.52€	50.52€	*Link_24*	Electronic
22	Wire	Red wire 1-mm^2^ section	1^6^	49.58€	49.58€	*Link_25*	Electronic
23	Wire	Black wire 0.5-mm^2^ section	1^6^	28.23€	28.23€	*Link_26*	Electronic
24	Wire	Red wire 0.5-mm^2^ section	1^6^	28.23€	28.23€	*Link_27*	Electronic
25	Battery	CR1225 lithium battery	1	6.54€	6.54€	*Link_23*	Electronic
26	Micro SD card	Micro SD Card 16 Go Class 10	1	6.99€	6.99€	*Link_6*	Electronic
27	Adapter	AC/DC 3 V Adapter	1	11.42€	11.42€	*Link_11*	Electronic
28	USB Cable	USB µUSB connector	1	2.46€	2.46€	*Link_8*	Electronic

Note: Links and prices are dated April 15, 2024.

^1^Sold by 250, but only 4 needed.

^2^Sold by 250, but only 6 needed.

^3^Sold by 100, but only 4 needed.

^4^Sold by 100, but only 6 needed.

## 5 Assembly instructions

### 5.1 Additional tools and resources required

• Socket and/or open-end wrenches
• Precision screwdriver set
• Needle-nosed pliers
• Wire stripper
• Electrical multimeter (for troubleshooting)

### 5.2 Data logger assembly

**Fig. 4:**
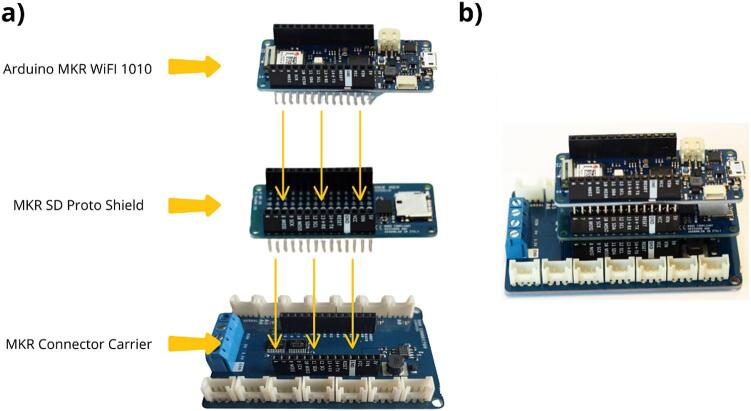
Assembly of Arduino boards, a) Assembly method of the MKR Connector Carrier, MKR SD Proto Shield and Arduino MKR Wi-Fi 1010 boards, b) Final result of the board assembly steps.

The first step consists of assembling the MKR CONNECTOR CARRIER with the MKR SD Proto Shield and the MKR Wi-Fi 1010 Arduino Board (Table 2, rows 1, 2 and 3). The MKR SD Proto Shield is mounted directly onto the MKR CONNECTOR CARRIER, with the MKR Wi-Fi 1010 board then stacked on top of the MKR SD Proto Shield, creating a compact three-layer assembly. The MKR Wi-Fi 1010 board is equipped with a microcontroller used to control and drive the data logger (Fig. 4 a). The MKR Proto SD card will store a data file and login history file on an SD card. Finally, the MKR Carrier card will act as the central platform connecting all the data logger components for the ultrasonic flowmeter. The Arduino MKR Wi-Fi 1010 board was chosen for:

• its Wi-Fi chip, which allows integration of an embedded web server and generating of a Wi-Fi access point in order to offer an interactive HMI;
• its compact size, low power consumption, modularity and European manufacturing.

This data logger proves to be very compact once assembled (Fig. 4 b).

**Fig. 5:**
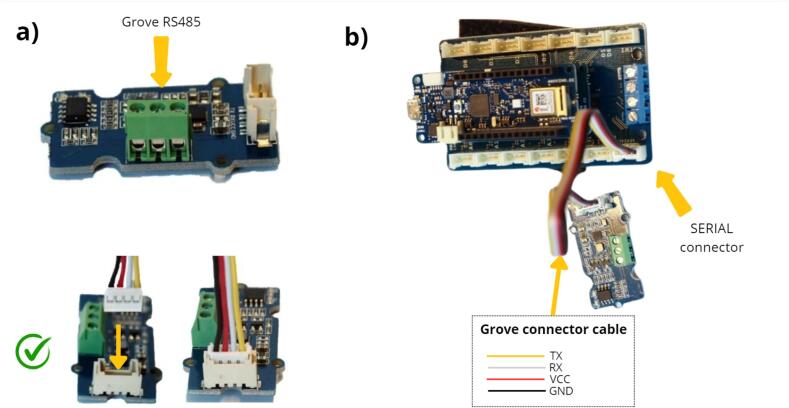
Installation of the Grove RS485 module, in order to communicate with the water-level sensor.

As shown in Fig. 5 a, the next step is to install the Grove RS485 module (Table 2, row 5). This module makes it possible to interface the sensors using the RS485 communication protocol, which has the triple advantage of allowing several RS485 sensors to be place (SERIAL connector) on the same bus, using long cable lengths of up to one kilometer, and providing good resistance to interference. This Grove module must be connected to the connector on the terminal board labelled SERIAL Connector, located on the MKR Connector Carrier.

**Fig. 6:**
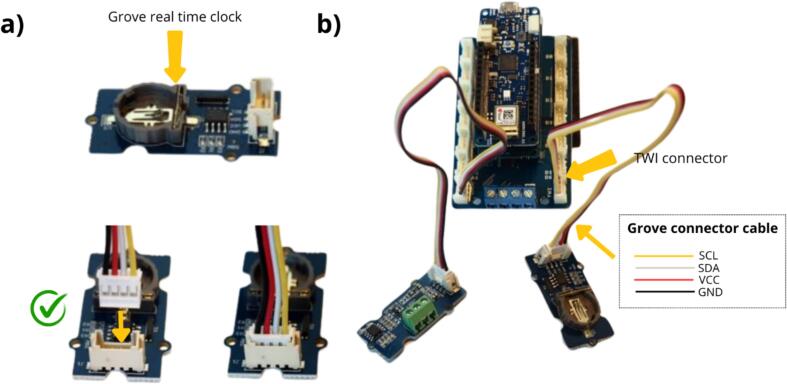
Installation of the Grove RTC module for time management purposes.

The next step is to install the Grove RTC module (Table 2, row 4), which uses an RTC integrated circuit that includes a real-time clock with a backup battery to maintain time even when the main power is cut off. This Grove module must be connected to the connector on the terminal board labelled TWI Connector, located on the MKR Connector Carrier (Fig. 6).

**Fig. 7:**
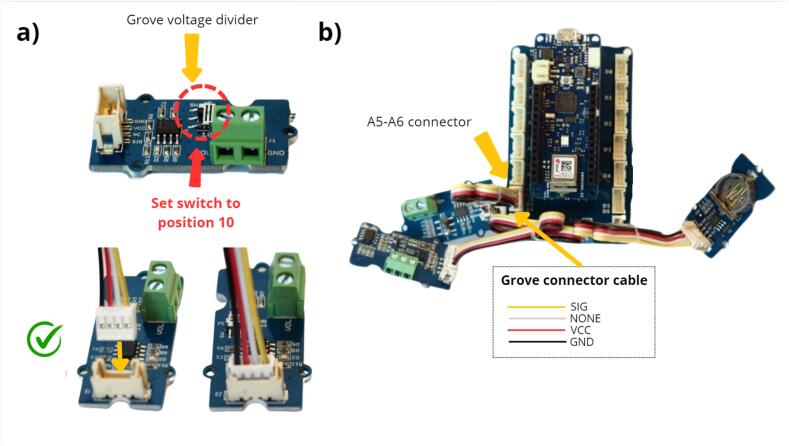
Installation of the Grove voltage divider module for downward scaling.

The Grove voltage divider (a) is an electrical component designed to scale down a high input voltage to a lower, more manageable level suitable for interfacing with the MKR WiFi 1010, which only accepts a range between 0 and 5 V for a voltage measurement (Table 2, row 6). This module is used to measure the 12 V supply voltage of the data logger. It is important to ensure that the switch is set to position 10. This particular configuration ensures that the voltage is divided by 10 and measured on a scale of 0–––1.2 V. Such a step is crucial to protect the microcontroller on the Arduino board. The Grove voltage divider is mounted on the A5 / A6 connector on the MKR Connector Carrier (Fig. 7 b).

**Fig. 8:**
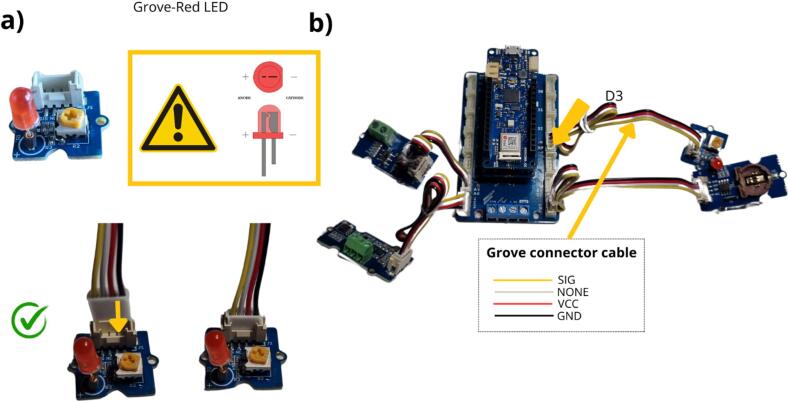
Installation of the Grove LED module for visual indication.

The Grove LED Module is a compact Grove compatible component designed to provide visual indication within the flowmeter design (Table 2, row 7 and Fig. 8 a). This module is plugged into the D3 connector, on the MKR Connector Carrier (Fig. 8 b). The LED provides the user with a number of indications, namely:

• The LED flashes rapidly to indicate that a program can be uploaded, then remains lit during system initialisation.
• The LED flashes once when the equipment is in measuring mode (one flash for each measurement).
• The LED remains on while the Wi-Fi is being activated.
• The LED stays off while the Wi-Fi is deactivated but the system is still functioning.

**Fig. 9:**
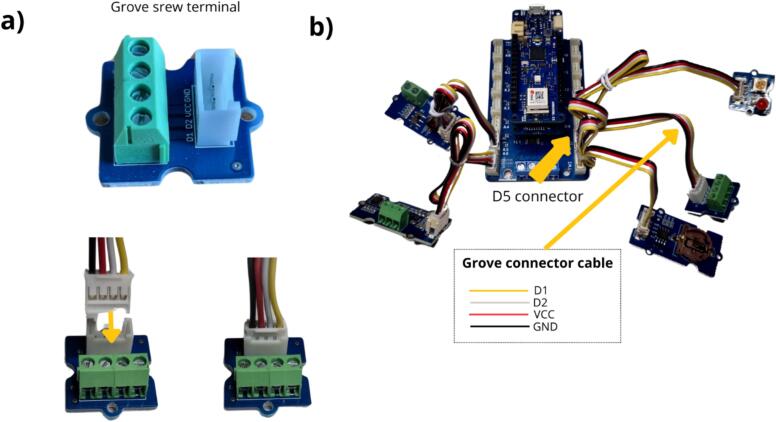
Installation of the Grove terminal screw module.

The final stage in assembling the data logger entails installing a Grove Terminal Screw (Table 2, row 8). This module will subsequently be used to connect a switch to activate the wireless functionality. It must be connected to port D5 of the MKR Connector Carrier (Fig. 9).

### 5.3 Data logger enclosure design

**Fig. 10:**
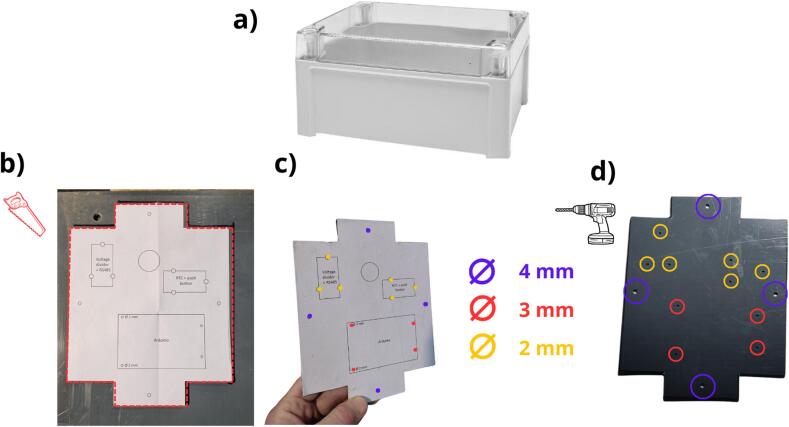
Preparation of the data logger enclosure: a) polycarbonate enclosure, b) PVC plate and template for cutting the plate and drilling, c) identification of drilling diameters, d) drilling of the PVC plate.

The next step consists of preparing the enclosure to receive the data logger. An IP65 polycarbonate box with a transparent cover has been selected for its ability to reveal the internal electronics and communication indicators.. (Fig. 10 a). A support plate is required to install the data logger inside the box. Two solutions for designing this plate are available:

• The first is to print (using a 3D printer) from a CAD file named CAD_3DPrint_support_flowmeter.STL, as presented in Table 1.
• The second involves the cutout and drilling of a 2–4 mm thick plate in the desired material (PVC, metal or wood).

**Fig. 11:**
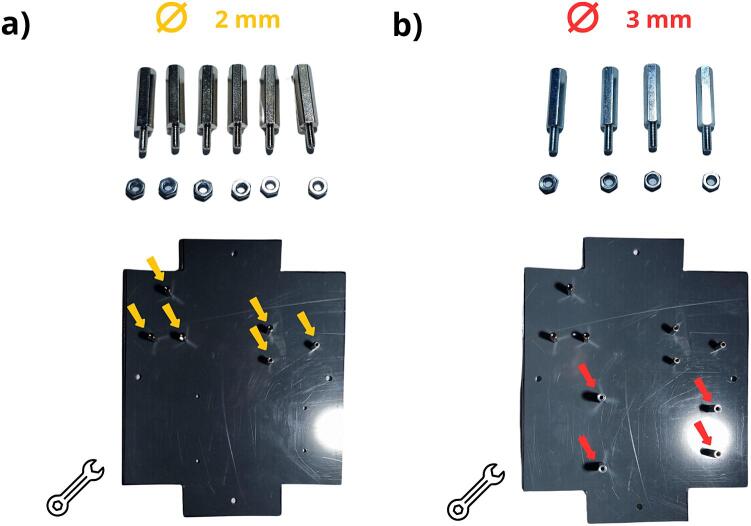
Installing spacers on the PVC plate: a) spacer M2 with a height of 2 cm, b) spacer M3 with a height of 2 cm.

**Fig. 12:**
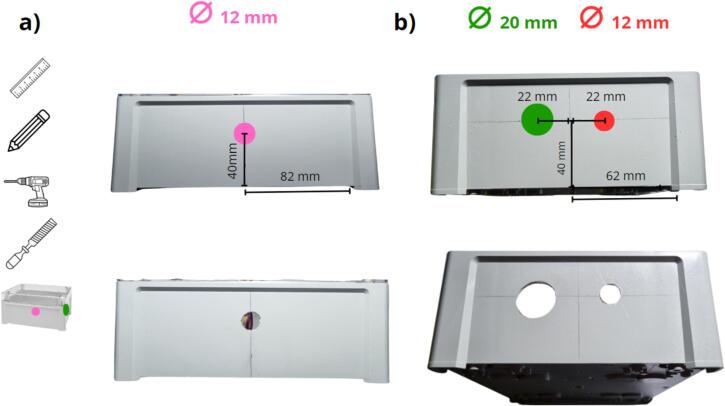
The holes are drilled on the data logger enclosure: a) 12  mm diameter holes on the long side, b) 20 and 12  mm diameter holes on the short side.

The PVC plate was cut out using the pattern (file: Flowmeter_data_logger_hole.pdf, indicated in Table 1. This pattern is printable at a 1:1 scale. The shape is to be cut out along the dotted lines, and then the sheet glued to the chosen material with paper glue (Fig. 10b). Next, the plate is cut and the holes drilled according to the pattern (Fig. 10 c and d), respecting the colour-coded diameters. After drilling, if paper glue has been used, the pattern can be removed by moistening the paper. Fig. 11 a and b show the installation of the spacers, which have been identified in rows 10 and 11 of Table 2. The spacers are fixed with M2 and M3 nuts (Table 2, rows 12 and 13). The subsequent step, as depicted in Fig. 12 a and b, consists of drilling the holes in the enclosure. The hole positions must therefore be marked on the enclosure and the holes drilled, both respecting the indications shown in the figure (positioning and diameters). After drilling, the holes can be deburred, if necessary.

**Fig. 13:**
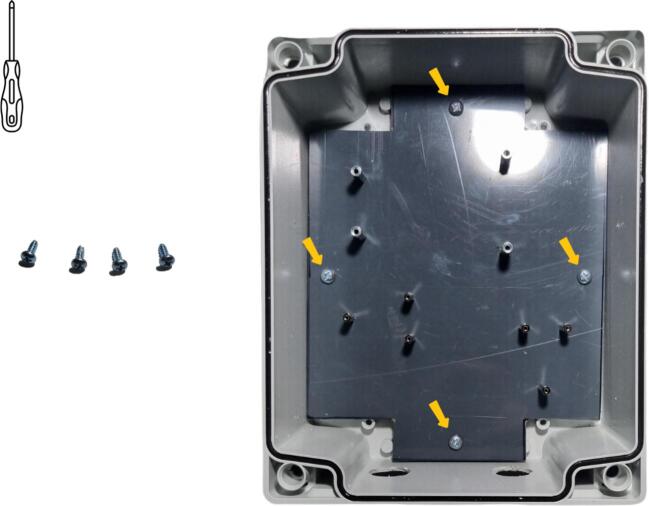
Installation of the PVC plate with spacers in the data logger box (this installation is the same for the 3D print support plate).

The final stage in preparing the enclosure entails installing the PVC plate in the enclosure. This installation, as shown in Fig. 13, makes use of the four M3 screws included with the polycarbonate enclosure, to attach the plate to the enclosure.

### 5.4 Mounting of the data logger, sensor and power supply

**Fig. 14:**
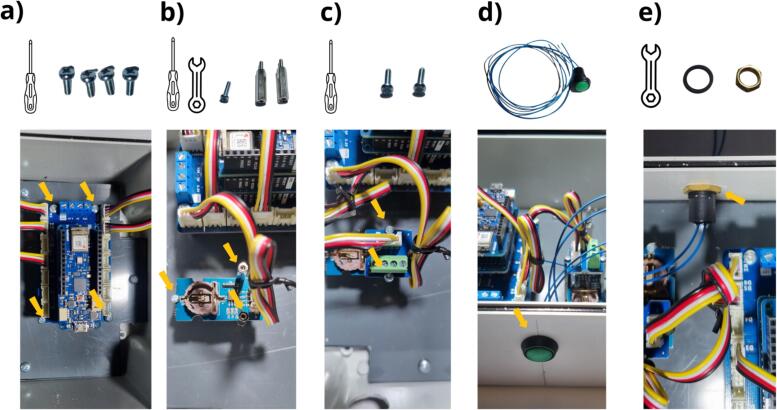
Mounting of the data logger in the enclosure: a) secure the Arduino MKR Connector Carrier in the enclosure using the M3 x 8  mm screw, b) install the RTC module onto the spacers with M2 x 8  mm screw and M2 x 20  mm spacers, c) install the Grove Screw Terminal module with M2 x 8  mm screws, d) install the pushbutton, e) screw the pushbutton seal and nut.

**Fig. 15:**
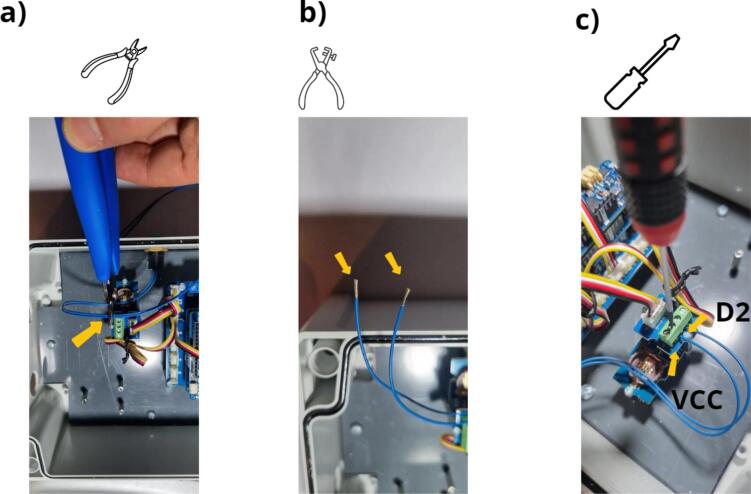
Connection of the pushbutton to the Grove Screw Terminal module: a) cut the wires to the appropriate length, b) strip the wires by 5 mm, c) fasten the wires to the two central screws of the Grove Screw Terminal.

To position all components on the plate, the reader is referred to the following figure: Flowmeter_data_logger_hole.pdf, which is available in the repository (Table 1). Data logger installation will begin with positioning of the data logger board and, in particular, installation of the Arduino MKR Connector Carrier in the box (Fig. 14 a). Four M3 x 8  mm screws must be used to attach it (Table 2, row 14) to the four M3 spacers (Table 2, row 11). For the following stage (Fig. 14 b), the Grove RTC module is attached to the three corresponding M2 spacers using one M2 x 8  mm screw (Table 2, row 15) and two M2 x 20  mm spacers (Table 2, row 10). Above the two columns used to attach the Grove RTC module, the Grove Screw Terminal Module can be mounted (Fig. 14 c) and secured by means of two M2 x 8  mm screws. The next step is to install and secure the pushbutton (Table 2, Row 16) in the 12  mm diameter hole (Fig. 14 d and Fig. 12 a). The pushbutton should now be connected to the Grove Screw Terminal module (Fig. 15). As shown in Fig. 15 a, the cables are to be cut to the correct length in order to reach the Grove Screw Terminal module. These cables should then be stripped to a length of 5 mm (Fig. 15 b). Lastly, the cables should be connected to the two central screws (VCC and D2) of Grove Screw Terminal module (Fig. 15 c).

**Fig. 16:**
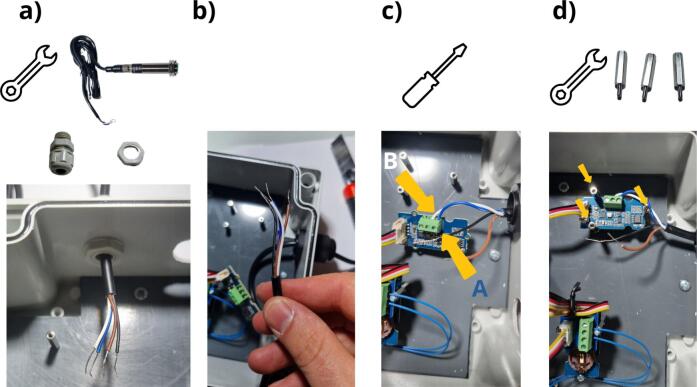
Installation of the Ultrasonic Sensor URM14 SEN0358: a) attaching the gland and installing the sensor wire, b) presenting the wires, c) connecting the RS485 module, d) attaching the RS485 module to the housing.

Fig. 16 shows the installation of the Ultrasonic Sensor (Fig. 16 and Table 2, row 17). The first step calls for installing the cable gland (Fig. 16 a and Table 2, row 18), in order to allow the wire to pass through the wall. Fig. 16 b shows the four wires of this sensor. It is now necessary to connect the blue (RS485-B) and white (RS485-A) wires to the Grove RS485 module (Fig. 16 c). The blue wire must be connected to terminal block A and the white wire to terminal block B. As a final step, the RS485 module is fastened on the three corresponding M2 spacers with 3 M2 x 20  mm spacers (Fig. 16 d and Table 2, row 10).

**Fig. 17:**
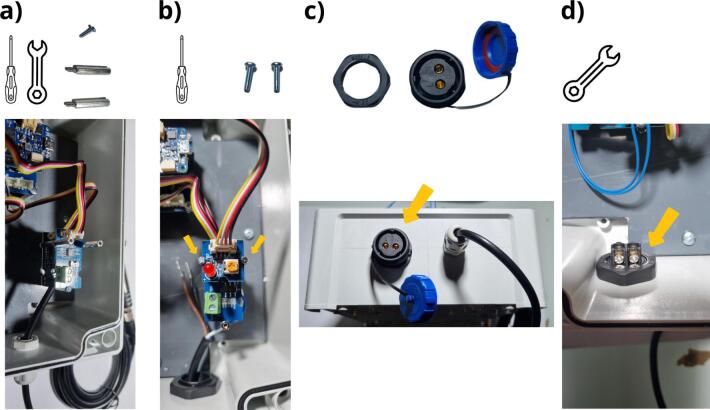
Installation of a) the voltage divider, b) the red LED grove module, c) the power supply female connector, d) Tightening of the nut to ensure connector attachment.

The Grove Voltage Divider module should then be positioned on top of the RS485 module (Fig. 17 a). This module is attached using a single M2 x 8  mm screw (Table 2, row 15) and two M2 x 20  mm spacers (Table 2, row 10) and the Grove Red LED module can be positioned above and attached with two M2 x 8  mm screws (Fig. 17 b). The power supply female connector (Table 2, row 19) is to be installed on the enclosure in the 20  mm diameter hole (Fig. 17 c), while the nut is to be screwed to ensure connector attachment (Fig. 17 d).

**Fig. 18:**
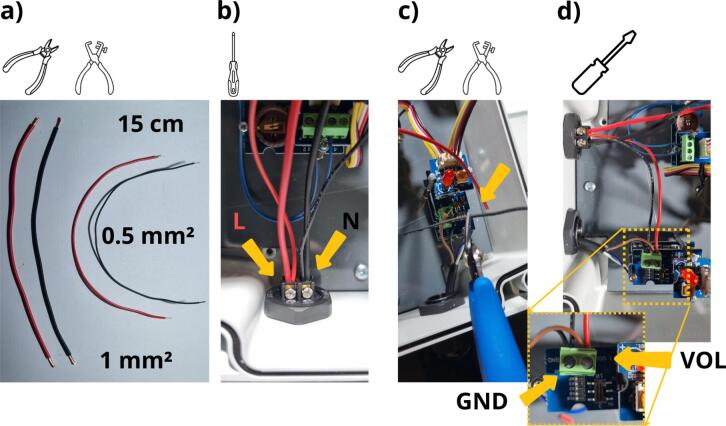
Connecting the power cables: a) preparing the cables, b) connecting the cables to the female connector, c) fitting the length of the cables, d) connecting the cables to the Grove Voltage Divider module.

To power the various elements, the several wires in the enclosure still need to be connected (Fig. 18). It is first necessary to cut one red wire and one black wire of 15  cm length and 1-mm^2^ cross-section (Table 2, rows 21 and 22) before cutting one red wire and one black wire of 0.5-mm^2^, also 15 cm long (Table 2, rows 23 and 24). The ends of the wires should be stripped (5 mm). Next, connect the two red wires to the L terminal and the two black wires to the N-terminal on the power supply socket (b). It then becomes necessary to adjust the length of the 0.5-mm^2^ black and red wires so as to reach the screw terminals of the Grove Voltage Divider module (Fig. 18 c). On this divider module, the brown wire of the ultrasonic sensor (VCC) and the 0.5-mm^2^ red wire are to be connected to the VOL terminal, while the black wire of the ultrasonic sensor (GND) and 0.5-mm^2^ black wire on the GND terminal (Fig. 18 d).

**Fig. 19:**
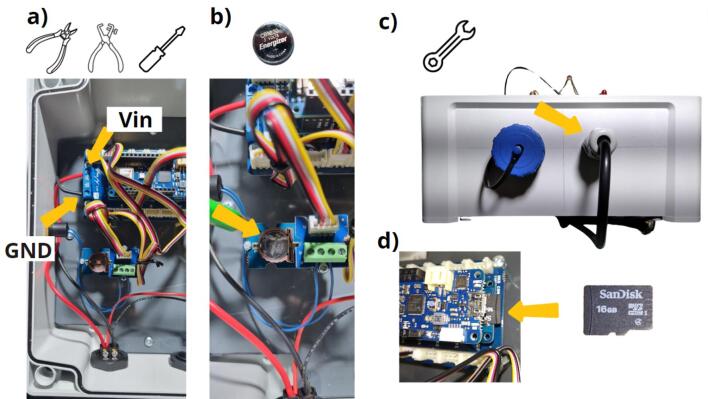
The final implementation step for the data logger: a) connect the red and black 1-mm^2^ cables to the Arduino MKR Carrier connection, b) introduce the CR1225 battery in order to power the Grove RTC module, c) screw the external nut of the gland, d) insert the SD card into the Arduino SD proto shield.

**Fig. 20:**
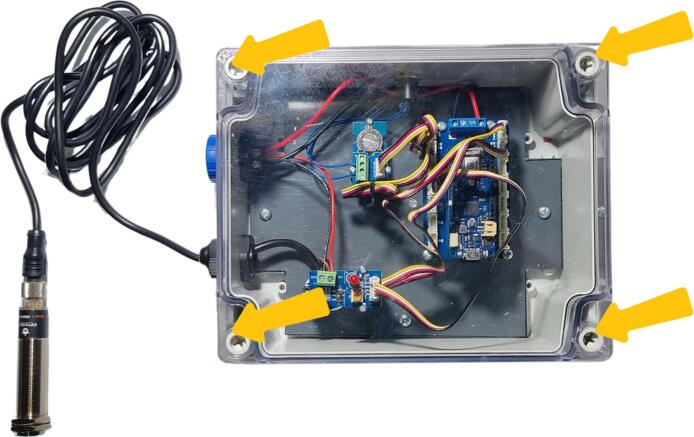
Flowmeter data logger enclosure.

The final steps of mounting the data logger are shown in Fig. 19. The black and red 1-mm^2^ cables still need to be connected to the Arduino MKR connector carrier (Fig. 19). The red wire needs to be connected to the Vin terminal block and the black wire to the GND terminal block. The CR1225 battery must then be installed in the Grove RTC module (Fig. 19, Table 2, row 25). On the outside of the housing, the cable gland nut still needs to be tightened (Fig. 19 c). Finally, the SD card can be inserted into the MKR SD proto Shield (Fig. 19, Table 2, row 26). It is recommended to use at least a HC1 class 10 SD card and format it in FAT32. The data logger is now completely assembled (Fig. 20).

### 5.5 Power supply and connectors

**Fig. 21:**
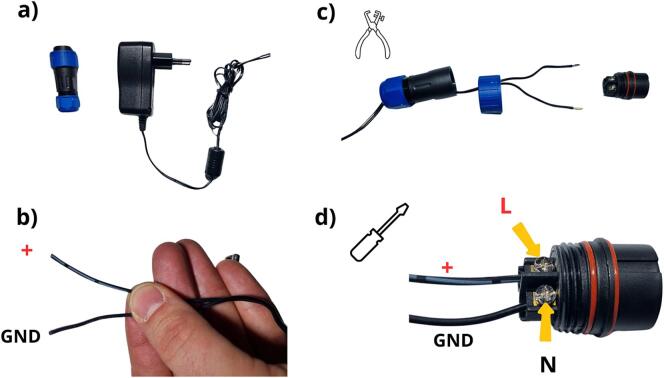
Preparation of the power supply system: a) power supply and connector, b) identification of the polarity of the power supply wires, c) opening of the connector and positioning of the cables, d) connection of the cables to the screw terminal block of the connector.

To power the data logger, a 12 V power supply must be used. In our case, we have opted for a 220 V-to-12 V converter. For this step, both connector (Table 2, row 20, Fig. 21 a) and power supply (Table 2, row 27, Fig. 21 a) must first be prepared. The original power connector supplied with the power supply must be cut off. The next step is to verify the polarity of the wires: the + is identifiable by small white lines written on the cable (Fig. 21 b). Then comes the step of stripping the ends of the cable (at around 5 mm) and opening the connector to pass the cable, as shown in Fig. 21 c. At this point, it is necessary to position the positive polarity wire into the L screw terminal block, and the ground wire (GND) into the N polarity screw terminal block (Fig. 21 d).

**Fig. 22:**
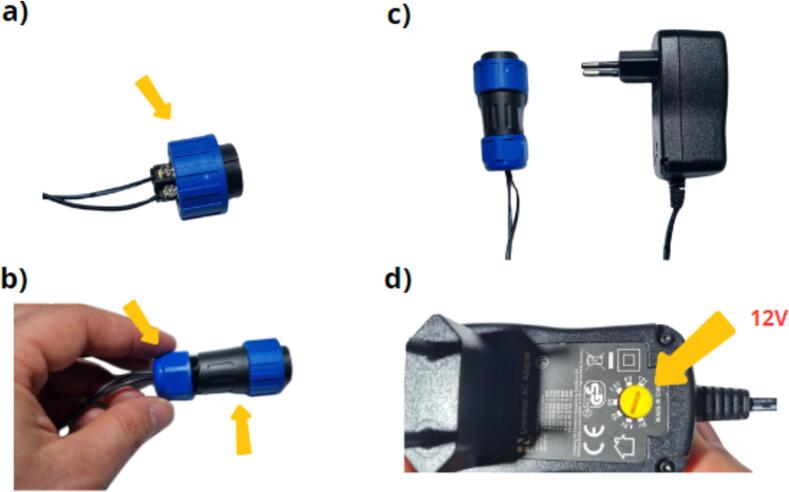
Finalisation of the power supply: a) installation of the clamping ring, b) installation of the body and nuts, c) assembly of the power supply and connector, d) adjustment of the power supply system to the 12  V position.

**Fig. 23:**
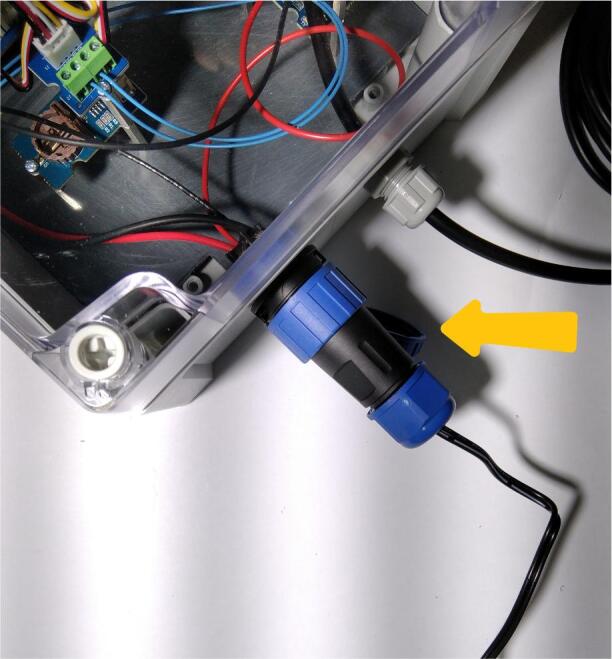
Connecting the power supply to the data logger.

Following the instructions given in Fig. 22 a, b and c, the connector and power supply assembly is complete, and the power supply connector can be closed. Before connecting the power supply to the data logger, the power supply system voltage must be set to 12 V and verified with a voltmeter (Fig. 22 d). The connector can now be plugged into the data logger power connector (Fig. 23).

## 6 Operating instructions

### 6.1 Programming: Arduino code description

Programming this data logger requires the Arduino IDE (Version 2.3.2, [23]) and the corresponding specific libraries:

- Arduino Low Power (Version 1.2.2, [24])
- Adafruit_BusIO (Version 1.16.1, [25])
- Wire (Arduino Core, [26])
- RTCLib (Version 2.1.4, [27])
- RTCZero (Version 1.6.0, [28])
- SD (Version 1.2.4, [29])
- SPI (Arduino Core, [30])
- WiFiNINA.h (Version 1.8.14, [31])
- FlashStorage (Version 1.0.0, [32])

The language used is C++. The system code is a folder containing five files with extension*.ino* and four files with extension*.h*. As this program is common to several types of data loggers (not presented in this paper), the Config_File.h will allow the program to be compiled according to the chosen data logger version. This file allows defining: the name assigned to the data logger and subsequently to the generated Wi-Fi, the measurement frequency, the type of data logger, and the characteristic heights of the monitored channel. All.*ino* files are common across all data logger types. The Config_File.h provided in the on-line repository comes pre-configured for flowmeter applications and only the HT_Eau.h and Config_File.h files will be taken into account during compilation. The other.h files will be ignored and not imported on the microcontroller.

**Fig. 24:**
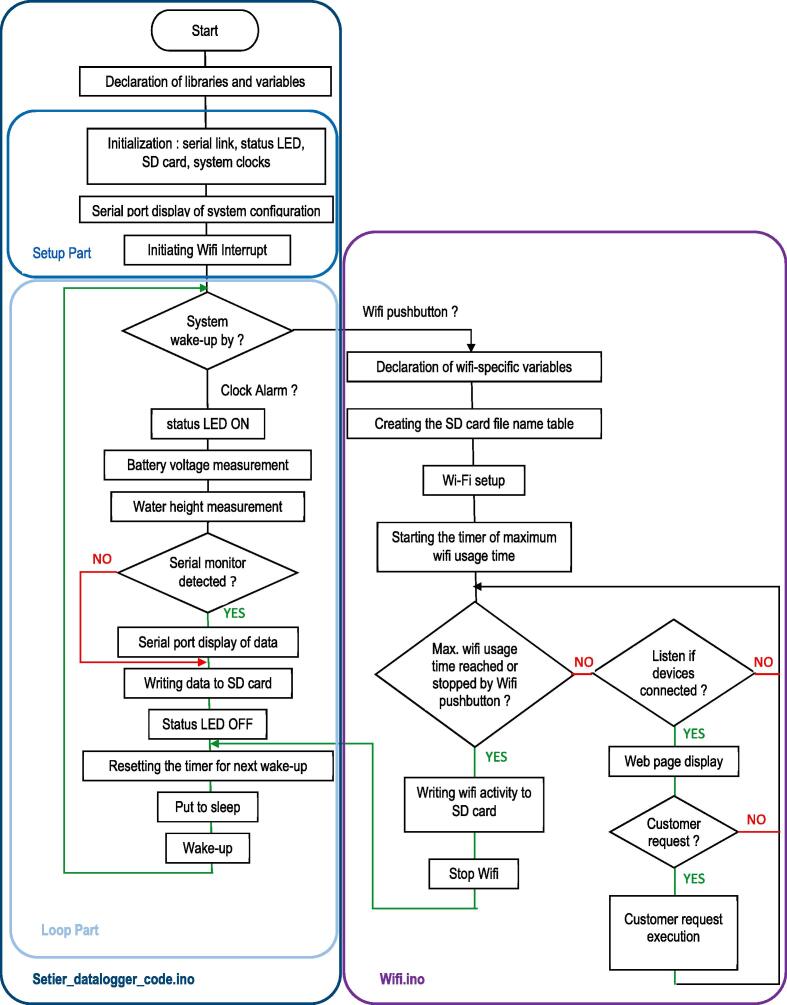
Flowmeter program flowchart.

The main program (Fig. 24) lies in the file named setier_datalogger_code.ino, and the other.ino file brings together subprograms for managing Real Time Clock (Base_RTC.ino), Wi-Fi (Base_Wifi.ino), SD card (Base_SD.ino) and reading battery voltage (Base_Vbatt.ino).

The main program starts with the list of libraries and variables used. The set-up part includes initialisation of the various system elements and calls up some initialisation subprograms. This part is executed only once, at the time of powering on. The loop part is executed each time the system wakes up, by pressing the button to activate the Wi-Fi (the subprogram Awake_Wifi in the tab Base_Wifi is executed) or by means of the Real Time Clock to carry out the measurement.

At the end of the measurement cycle, data are written to the SD card, and the system goes back to sleep. Subprograms for measurement and data written to the SD card can be found in HT_Eau.h. The last two subroutines of the main file are interrupting subroutines and make it possible to identify the cause of the system waking up.

The only file that needs to be modified before loading the code on the Arduino board is Config_File.h. By default, all lines are commented expected the following lines:

1. *define STATION_NAME “Flowm”* − used to name the data logger, this name may be changed, but must not exceed 8 characters;
2. *define Ma_Cadence* chooses the measurement frequency *defines TYPE_DATA LOGGER_HAUTEUR_EAU 1* − an option defining the type of data logger;
3. *define Ht_Debit_NUL 700* − value used to define the zero-flow head in relation to the position of the ultrasonic sensor, in this case 700 mm above the position of the ultrasonic sensor;
4. *define Profondeur_Canal 300* − possibility to enter the maximum variation of the water level up to the zero-flow head.

To load code onto the data logger, plug the power supply into a socket, then connect the USB cable (Table 2, row 28) between computer and data logger. To use the Arduino IDE to manage the code, please refer to the corresponding Arduino documentation (https://docs.arduino.cc/).

### 6.2 Startup procedure

The following steps must be followed:

1. When the program is configured, transfer it on Arduino board.

2. Open the serial monitor on Arduino IDE.

3. Check that the red LED (on Grove module) flashing rapidly during initialisation.

4. The data logger sends its configuration to the serial monitor, check it, modify the config file, if the configuration is not good and restart the procedure.

5. The data logger sends its first measurement to the serial monitor, checking the time and distance measurement.

6. The USB cable can be disconnected and the enclosure closed.

### 6.3 Wi-Fi connection

The data logger is now operational. It can be left running for a few minutes to acquire a certain amount of data. From here, simply follow the procedure below to connect your smartphone to your data logger:

1. Activate Wi-Fi on your phone;
2. Turn mobile data off;
3. Press the green button on the data logger (Fig. 14 c);
4. Connect to the Wi-Fi emitted by the data logger, it will show the name selected in the Config_File.h;
5. Go to a browser and enter the following IP address: 192.168.4.1;
6. A webpage opens if connections to http pages are allowed (no restriction on https pages);
7. Check the name and date of the data logger;
8. Carry out a measurement by clicking “update”;
9. Download the data file by clicking the txt file name;
10. Shut off the Wi-Fi by once again pressing the green button or else by waiting 15 min: the Wi-Fi will automatically be disconnected after this time.

It is important to note that when the Wi-Fi is activated, no measurement is acquired and saved on the SD card.

### 6.4 Shutdown procedure

No special procedure exists for turning off the data logger. Just be mindful of the completion of the measurement (red LED must be off) and simply disconnect the power supply.

## 7 Validation and characterisation

### 7.1 Comparison with a reference flowmeter

**Fig. 25:**
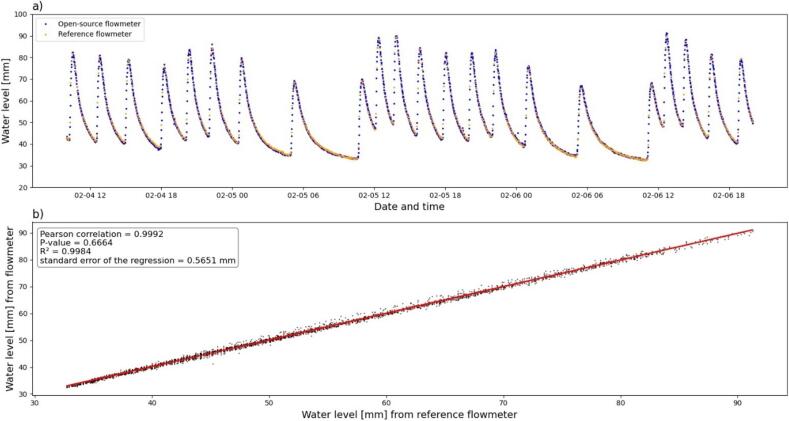
Comparison of the open source flowmeter with the reference flowmeter: a) water head measurement for the two flowmeters, b) correlation between both flowmeters.

To validate our flowmeter, we first sought to compare our ultrasonic water height measurement with an industrial reference bubble measurement (BubleFLO2, HYDREKA brand) that also measures the water level. We chose the BubbleFLO2 measurement to avoid any disturbances potentially occurring with another ultrasonic flowmeter. The two devices were installed at the outlet of a wastewater treatment plant located 40 km from Lyon (France). This 1,000-PE treatment plant is equipped with an ISMA type II Venturi channel (up to 200 m^3^.h^−1^). We have only presented herein the water-level measurements without conversion into flow over a two-day period to show only the raw signals. Fig. 25 a shows the measurements obtained with the reference flowmeter and open source flowmeter: the two curves overlap perfectly. Fig. 25 b demonstrates that the correlation between the two measurements highlights a relevant correlation criterion and standard regression error of 0.6 mm compared with the reference flowmeter. The open source flowmeter also provides a reproduction of water-level variations.

### 7.2 Long-term monitoring

**Fig. 26:**
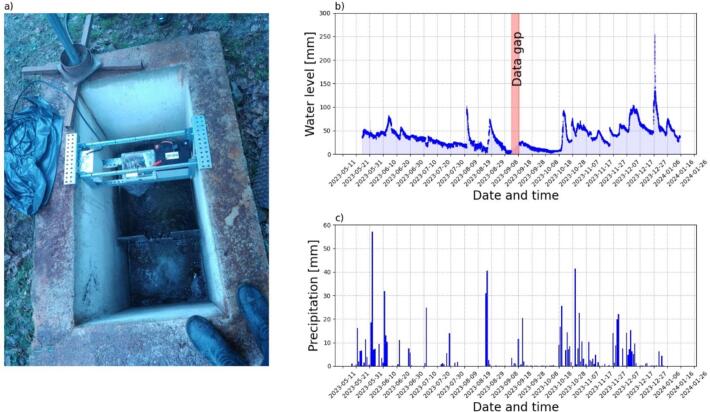
Long-term installation of an ultrasonic flowmeter on a wastewater treatment plant outlet: a) photo of the installation, b) results of water level monitoring, c) precipitation measured near the wastewater treatment plant.

Fig. 26 shows the results of a long-term installation at a wastewater treatment plant. The treatment facility is equipped with a rectangular weir (Fig. 26a). The ultrasonic sensor is positioned in the centre of the channel and the Fig. 26 b shows the water level monitoring, which was operating well over 10 months from May 2023 to January 2024. The selected treatment plant has a network that collects high levels of rainwater. Hence, when rainfall occurs in the sector, the flowmeter often observes an increase in water level.

## 8 Conclusion

The two experiments proposed here demonstrate the validity of our ultrasonic flowmeter manufactured using a low-tech data logger and a low-cost sensor. The metrological characteristics evaluated are in line with user requirements and the different measurement conditions found in wastewater treatment plants. The step-by-step design guide is intended to encourage innovation so that anyone, even without a background in electronics, can contribute to the development of low-tech applications for researchers and operators. In particular, its solderless design makes it modular and allows total control over the design of the device to adapt it to the local context in which it will be installed. This greatly improves the repairability of the flowmeter and therefore its long-term use, reducing its impact on the environment. The device could also be applied to other field configurations, such as urban hydrology, thereby increasing its deployment rate and the number of potential users.

## 9 Ethics statement

The authors have nothing to declare under this heading.

## CRediT authorship contribution statement

**Hélène Guyard:** Writing – original draft, Validation, Software, Methodology, Investigation, Data curation, Conceptualization. **Stéphanie Prost-Boucle:** Writing – review & editing, Validation, Supervision, Methodology, Funding acquisition. **Julien Sudre:** Writing – review & editing, Validation, Software, Resources, Methodology, Investigation, Formal analysis, Data curation, Conceptualization. **Sylvain Moreau:** Writing – review & editing, Validation, Methodology, Formal analysis, Conceptualization. **Arnold Imig:** Software, Resources, Methodology, Conceptualization. **Gabrielle Favreau:** Writing – review & editing, Validation. **Valerie Quatela:** Writing – review & editing. **Remi Clement:** Writing – original draft, Visualization, Validation, Supervision, Funding acquisition, Formal analysis, Data curation, Conceptualization.

## Declaration of competing interest

The authors declare that they have no known competing financial interests or personal relationships that could have appeared to influence the work reported in this paper.
