# Supplementary figures and images for: An open source ultrasonic flowmeter for monitoring the input/output flow rates of wastewater treatment plants
Source: HardwareX. 2024 Dec 11;21:e00613. doi: 10.1016/j.ohx.2024.e00613 (PMC11719284; doi:10.1016/j.ohx.2024.e00613)

# Flowmeter\_datalogger\_hole.pdf

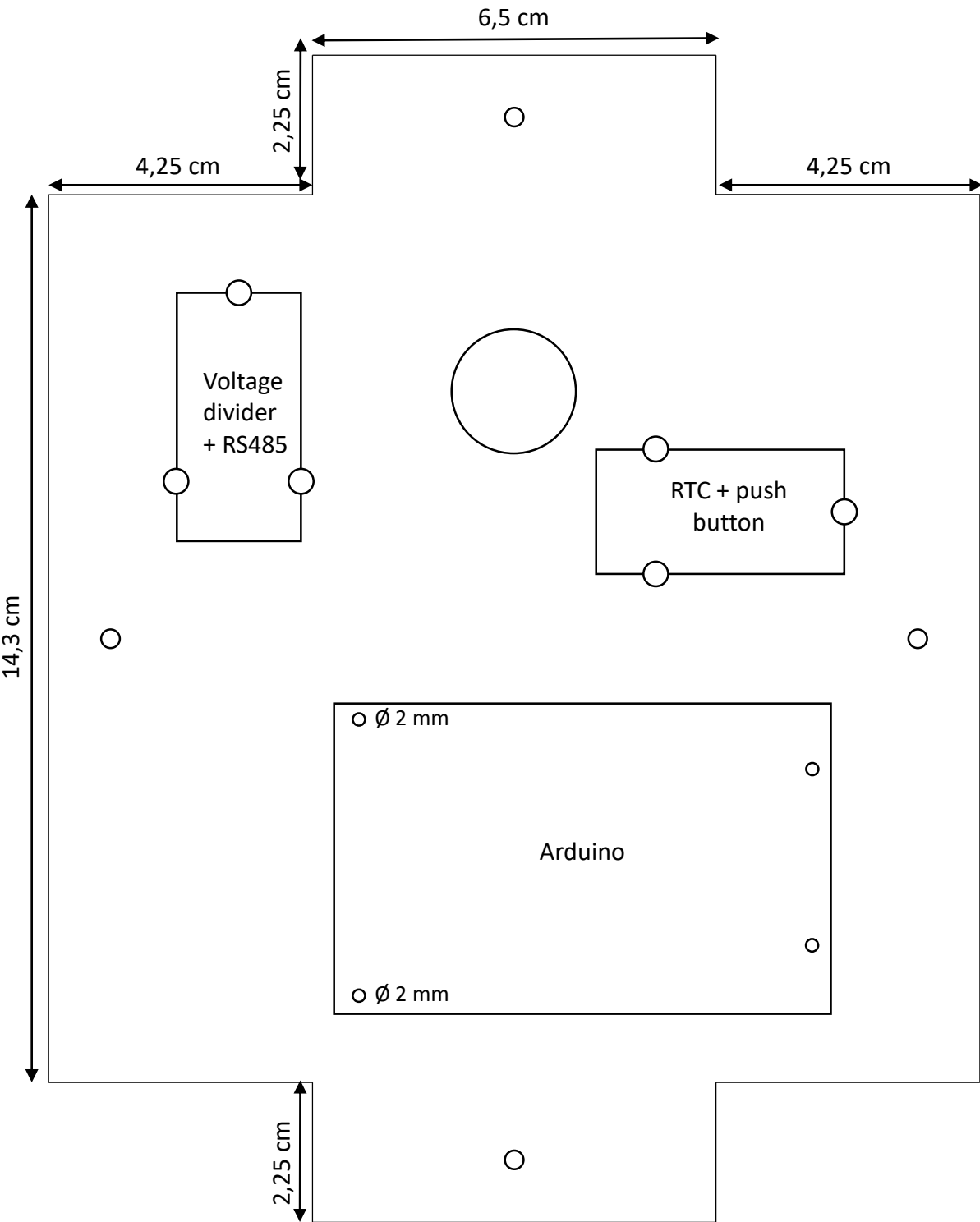

Supplement: Supplementary Data 1 [file mmc1.pdf]
